# ECM remodeling in hypothyroidism-associated MAFLD: mechanisms, clinical relevance, and therapeutic targets

**DOI:** 10.3389/fimmu.2025.1639196

**Published:** 2025-10-03

**Authors:** Tianzhen Wang, Liyun Duan, Beiying Zhao, Jingbin Zhang, Yongjiang Yu, Jinyue Zhao

**Affiliations:** ^1^ College of Traditional Chinese Medicine, Changchun University of Chinese Medicine, Changchun, China; ^2^ The First Clinical Medical College, Shandong University of Traditional Chinese Medicine, Jinan, China; ^3^ Breast and Thyroid Surgery, Jilin Province People’s Hospital, Changchun, China; ^4^ The Affiliated Hospital to Changchun University of Chinese Medicine, Changchun, China

**Keywords:** hypothyroidism, MAFLD, ECM remodeling, HSC, TH signaling, liver fibrosis

## Abstract

Metabolic dysfunction–associated fatty liver disease (MAFLD) is a major chronic liver disease increasingly linked to endocrine and immunometabolic dysregulation. Hypothyroidism, both overt and subclinical, has emerged as a significant endocrine risk factor for MAFLD. Extracellular matrix (ECM) remodeling, a hallmark of fibrosis, represents a crucial but underexplored mediator in this process. This review highlights how altered thyroid hormone (TH) and thyroid-stimulating hormone (TSH) signaling promote ECM remodeling through metabolic, inflammatory, and fibrogenic pathways, with emphasis on hepatic stellate cell (HSC) activation and immune reshaping. We further summarize ECM-derived biomarkers and emerging therapeutic strategies, including THRβ agonists and ECM-targeted approaches.

## 1 Introduction

Metabolic dysfunction–associated fatty liver disease (MAFLD) affects nearly one in four individuals globally and constitutes a major contributor to liver-related morbidity and mortality (1). Thyroid dysfunction, particularly hypothyroidism, is increasingly recognized as a comorbidity that may exacerbate hepatic steatosis, inflammation, and fibrosis (2). Epidemiological and meta-analytic studies indicate that even subclinical hypothyroidism is associated with higher MAFLD prevalence, greater disease severity, and increased fibrosis risk, particularly among obese and postmenopausal populations (3–6). Elevated TSH levels correlate with hepatic steatosis, advanced fibrosis, and hepatocellular carcinoma risk (7, 8). Despite these associations, the mechanistic links between hypothyroidism and MAFLD remain incompletely defined. Emerging evidence implicates extracellular matrix (ECM) remodeling as a central mediator bridging endocrine dysfunction and hepatic injury (9). The ECM not only provides structural support but also modulates cell signaling, thereby influencing hepatocyte survival, insulin sensitivity, and immune homeostasis (10, 11). In MAFLD, aberrant ECM remodeling is characterized by excessive fibrillar collagen deposition, increased tissue stiffness, and dysregulated turnover of matrix-associated proteins and glycosaminoglycans, including hyaluronan, as well as receptors such as RHAMM, CD44, and integrins (12, 13). These alterations promote fibrosis and metabolic dysregulation by modifying the hepatocyte microenvironment and regulating growth factor and cytokine signaling (14–17).

In this review, we synthesize clinical and experimental evidence to delineate the role of ECM remodeling in hypothyroidism-associated MAFLD. We focus on endocrine regulation of ECM dynamics, the crosstalk among hepatic stellate cells (HSCs), immune cells, and the fibrotic niche, and integrin-mediated mechanotransduction pathways linking matrix stiffness to pro-fibrotic gene expression (18). Finally, we discuss ECM-derived biomarkers and potential therapeutic strategies, including thyroid hormone receptor β (THRβ) agonists and integrin inhibitors, emphasizing ECM remodeling as both a mechanistic and therapeutic nexus in hypothyroidism-driven MAFLD.

## 2 Thyroid dysfunction and MAFLD: pathophysiological links

MAFLD, previously referred to as nonalcoholic fatty liver disease (NAFLD), is a redefined entity that encompasses hepatic steatosis in conjunction with metabolic dysregulation—such as obesity, type 2 diabetes mellitus, or other metabolic risk factors (19, 20). This updated terminology more accurately reflects the disease’s underlying pathophysiology and emphasizes its close relationship with metabolic dysfunction. Therefore, the term MAFLD is adopted throughout this article (21). In hypothyroid states, triglyceride metabolism is markedly impaired, leading to hepatic triglyceride accumulation and elevated levels of circulating free fatty acids (8). When the liver’s metabolic capacity for primary energy substrates, including carbohydrates and fatty acids is exceeded, toxic lipid intermediates accumulate, inducing hepatocellular stress, injury, and death (22). These pathophysiological events contribute to hepatic insulin resistance (IR), altered gut microbiota composition, and a range of deleterious cellular responses, including mitochondrial dysfunction, endoplasmic reticulum (ER) stress, oxidative stress, and excessive production of reactive oxygen species (ROS). The sustained presence of these insults drives chronic hepatic inflammation and ultimately promotes MAFLD progression (23, 24).

### 2.1 Thyroid hormones and receptors in MAFLD: metabolic and fibrotic regulation

Building upon the mechanistic links between hypothyroidism and MAFLD, this section highlights the roles of THs and their nuclear receptors in regulating hepatic metabolism and fibrogenesis. Triiodothyronine (T3), the active form of TH, enhances lipolysis and promotes weight loss, whereas reduced T3 levels are associated with impaired lipid degradation, decreased cholesterol clearance, and increased adiposity (25). These metabolic effects are primarily mediated by thyroid hormone receptors (THRs), ligand-dependent nuclear transcription factors. In contrast, thyroxine (T4) binds THRs with approximately tenfold lower affinity, and its direct biological effects remain less well defined. The two main THR isoforms, THRα and THRβ, are encoded by separate genes. THRα is predominantly expressed in the heart, skeletal muscle, and HSC, while THRβ is highly expressed in hepatocytes and also found in the brain, kidney, and retina (26). Functional inactivation of THRα has been shown to enhance fibrogenic activity in HSCs (27). THRβ signaling plays a liver-specific role in regulating key metabolic pathways, including *de novo* lipogenesis, fatty acid β-oxidation, mitophagy, and cholesterol homeostasis. These processes collectively contribute to reductions in circulating low-density lipoprotein (LDL), apolipoprotein B (ApoB), and lipoprotein(a) [Lp(a)]. While T3 and T4 primarily act on hepatocytes to regulate lipid metabolism, THRα expression in HSCs suggests that excessive TH levels may activate these cells, potentially contributing to fibrogenesis in hyperthyroid states (28).

Growing evidence from clinical and experimental studies has established a significant association between hypothyroidism—both overt and subclinical—and the development and progression of MAFLD in both adult and pediatric populations (3, 29). Furthermore, hypothyroid states have been linked to greater risk and severity of liver fibrosis in MAFLD (30–33). In THR-deficient mouse models, spontaneous hepatic collagen accumulation and upregulation of fibrotic gene expression have been observed, highlighting the essential regulatory role of TH in fibrogenesis. Mechanistically, TH have been shown to suppress hepatic fibrosis by inhibiting SMAD2/3 phosphorylation within the TGF-β signaling pathway, thereby downregulating fibrosis-associated gene expression (27). Recent studies also implicate TSH in the pathogenesis of metabolic liver diseases (34). THs are critical regulators of organogenesis, cellular differentiation, and systemic metabolic homeostasis. Disruption of TH and TSH signaling has been associated with a range of liver disorders, including alcoholic liver disease, MAFLD, and hepatocellular carcinoma (HCC). In experimental models of carbon tetrachloride (CCl_4_)–induced liver injury, exogenous TH administration reduced the expression of key fibrogenic genes such as Col1a1 and Col1a2 (35).

Preclinical and clinical studies collectively support a protective role of thyroid hormones in liver homeostasis, underscoring the therapeutic potential of TH analogs in MAFLD management (36). Administration of T3 or synthetic TH analogs has demonstrated efficacy in attenuating hepatic steatosis and HCC development in rodent models exposed to high-fat diets or hepatocarcinogenic agents (4, 37–41). Additionally, TSH signaling has been shown to modulate hepatic lipid metabolism through its receptor on hepatocytes. Specifically, TSH induces hepatic steatosis by activating sterol regulatory element-binding protein (SREBP) pathways, inhibits bile acid synthesis by suppressing the SREBP2–hepatocyte nuclear factor 4 (HNF4)–CYP7A1 axis, and attenuates cholesterol biosynthesis via AMP-activated protein kinase (AMPK)–dependent phosphorylation of 3-hydroxy-3-methylglutaryl-coenzyme A reductase (HMGCR) (42). Together, these findings establish TSH as an independent regulator of hepatic lipid and cholesterol homeostasis.

### 2.2 Pathogenic mechanisms underlying hypothyroidism-associated MAFLD

#### 2.2.1 Simple steatosis: lipid accumulation and impaired lipophagy

Hypothyroidism promotes hepatic lipid accumulation through impaired lipid catabolism, enhanced *de novo* lipogenesis, and defective autophagic clearance of lipid droplets. In hypothyroid states, insulin receptor substrate (IRS)-mediated PI3K(phosphatidylinositol 3-kinase)/Akt(protein kinase B) signaling is attenuated, reducing insulin sensitivity and increasing hepatic glucose output (43–47). Decreased IRS2 expression and diminished Akt phosphorylation, observed in both hypothyroidism and NASH, directly link TH deficiency to hepatic insulin resistance (48, 49). Elevated circulating TSH exacerbates this process by activating toll-like receptor 4 (TLR4), c-Jun N-terminal kinase (JNK), and nuclear factor-κB (NF-κB) pathways, which inhibit IRS tyrosine phosphorylation and enhance serine phosphorylation, thereby aggravating insulin resistance (50, 51). However, these hypothyroidism-specific mechanisms remain less firmly established in clinical settings, as most supporting evidence derives from animal models and cross-sectional studies rather than longitudinal cohorts. Epidemiological studies support the association between hypothyroidism and insulin resistance, but evidence directly connecting this axis to ECM remodeling in MAFLD patients is currently insufficient.

In parallel, hypothyroidism disrupts systemic lipid metabolism by suppressing lipoprotein lipase (LPL) activity, impairing cholesterol efflux, and reducing triglyceride clearance (52, 53). These defects lead to elevated LDL-C(low-density lipoprotein cholesterol), total cholesterol, and intrahepatic triglyceride accumulation, reinforcing steatosis and lipotoxicity (54–56). Thyroid hormones also regulate hepatic autophagy, a key pathway for lipid degradation (lipophagy). Under physiological conditions, T3 activates AMPK and inhibits mTOR to sustain autophagic flux, inducing ULK1, Beclin-1, and LC3 transcription, while stimulating lysosomal biogenesis via TFEB(transcription factor EB) (57–59). Preclinical studies show that in hypothyroidism, reduced T3 availability suppresses AMPK activity, disinhibits mTOR, and diminishes autophagic flux, leading to impaired lipophagy and mitophagy (50, 60, 61). Current understanding of autophagy impairment largely relies on preclinical models, and direct evidence substantiating similar alterations in human hypothyroidism-associated MAFLD is still insufficient. These defects accelerate lipid accumulation, oxidative stress, and hepatocellular injury. Moreover, reduced Dio1(deiodinase type 1) expression in advanced MAFLD further exacerbates intrahepatic T3 deficiency, intensifying autophagy impairment (51, 62). Collectively, these mechanisms illustrate how hypothyroidism predisposes the liver to steatosis and metabolic dysfunction.

#### 2.2.2 Steatohepatitis (MASH): oxidative stress, inflammation, and immune activation

The progression from simple steatosis to steatohepatitis is critically mediated by oxidative stress and inflammation. Oxidative stress, largely derived from mitochondrial β-oxidation, induces hepatocellular injury and ECM remodeling (63). Patients with hypothyroidism display elevated markers of oxidative damage, and TSH directly stimulates reactive oxygen species (ROS) production (64). In MAFLD, oxidative imbalance is characterized by lipid peroxidation, depletion of polyunsaturated fatty acids, and dysregulation of SREBP-1c(sterol regulatory element-binding protein-1c) and PPAR-α(peroxisome proliferator-activated receptor alpha), alongside reduced nuclear factor erythroid 2–related factor 2(Nrf2) activity and diminished expression of detoxifying enzymes such as Nqo1 and HO1 (65–67). Preclinical studies demonstrate that hypothyroidism suppresses Nrf2 signaling, thereby amplifying oxidative injury and accelerating disease progression (68, 69). Under physiological conditions, T3 activates AMPK, the unfolded protein response (UPR), and autophagy to mitigate oxidative stress; these cytoprotective mechanisms are blunted in hypothyroidism (70, 71). Nevertheless, most evidence comes from animal studies and small-scale clinical observations, while large-scale prospective cohort validation is lacking. Large prospective cohorts and mechanistic human investigations are therefore required to validate this link.

In parallel, hypothyroidism reshapes the hepatic immune microenvironment. Elevated TNF-α and IL-1β, together with reduced adiponectin, promote NF-κB–dependent transcription of proinflammatory genes (72–75). Lipotoxic hepatocyte injury releases damage-associated molecular patterns (DAMPs) that activate Kupffer cells and recruit monocytes, which secrete cytokines (TNF-α, IL-6, CCL2) to amplify inflammation and stimulate hepatic stellate cells (HSCs) via TGF-β1 and PDGF (76–78). DAMPs and ROS also trigger NLRP3 inflammasome activation in Kupffer cells and recruited macrophages, leading to caspase-1–dependent cleavage of pro–IL-1β and pro–IL-18 into their active forms, thereby amplifying the inflammatory cascade. Activated HSCs perpetuate immune infiltration through chemokines such as CCL2, CCL5, IL-8, and CXCL12 (79), while neutrophils exacerbate hepatocellular damage by releasing myeloperoxidase (MPO) and ROS (80). Elevated TSH further augments TLR4/NF-κB signaling, reinforcing a vicious cycle between inflammation and insulin resistance (73, 81, 82). Together, these mechanisms converge to promote hepatocellular injury, immune cell infiltration, and HSC activation—hallmarks of MASH. But the specific contribution of hypothyroidism to these inflammatory pathways is supported mainly by experimental and associative data, with limited longitudinal clinical validation.

#### 2.2.3 Fibrosis and cirrhosis: ECM remodeling and sustained HSC activation

In advanced stages of MAFLD, hypothyroidism accelerates fibrosis progression through both direct and indirect mechanisms. Thyroid hormone (TH) deficiency compromises mitochondrial function, resulting in ATP depletion, membrane depolarization, and excessive ROS production (81, 82). Concomitant mitochondrial DNA damage, defective mitophagy, and reduced oxidative phosphorylation further exacerbate steatosis and drive fibrogenesis (70, 71, 83).

Under physiological conditions, triiodothyronine (T3) maintains mitochondrial integrity by inducing PGC1-α, Nrf1/Nrf2, and TFAM to stimulate mitochondrial biogenesis and fatty acid oxidation, while also activating AMPK signaling via integrin αvβ3 (84, 85). Loss of these protective pathways in hypothyroidism enhances oxidative stress and promotes fibrotic remodeling. Although mitochondrial impairment is consistently observed in both hypothyroidism and MAFLD, direct evidence linking thyroid dysfunction to ECM remodeling through mitochondrial pathways remains limited, and current understanding is based primarily on preclinical studies.

TH deficiency also exerts a direct influence on hepatic stellate cell (HSC) biology. TH receptor α (THRα), expressed on HSCs, mediates antifibrotic signaling, and its loss favors persistent HSC activation. This state is characterized by increased collagen I and α-SMA expression, as well as disruption of the MMP/TIMP balance, culminating in pathological ECM accumulation. Activated HSCs further engage mechanotransduction pathways, particularly integrin-mediated signaling, which amplify matrix stiffness and reinforce the fibrotic cascade.

**Figure 1:**
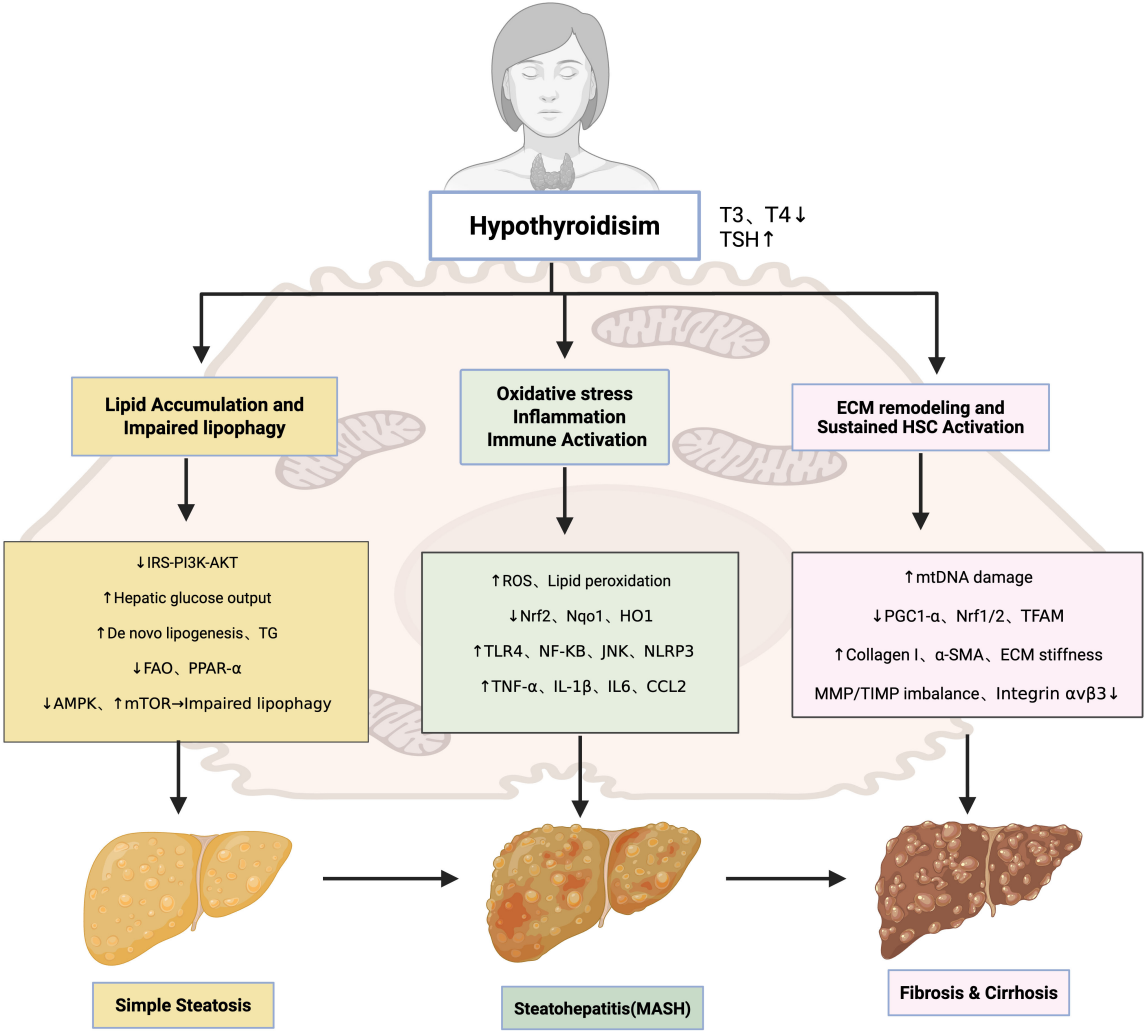
Pathogenic mechanisms underlying hypothyroidism-associated MAFLD.

Collectively, hypothyroidism facilitates the transition from steatohepatitis to fibrosis and cirrhosis by impairing mitochondrial homeostasis, suppressing antifibrotic THRα signaling in HSCs, and driving maladaptive ECM remodeling. These interconnected mechanisms are summarized in **Figure 1**, which illustrates the stepwise contribution of hypothyroidism to MAFLD progression.

## 3 ECM remodeling in hypothyroidism–MAFLD crosstalk

**Figure 2:**
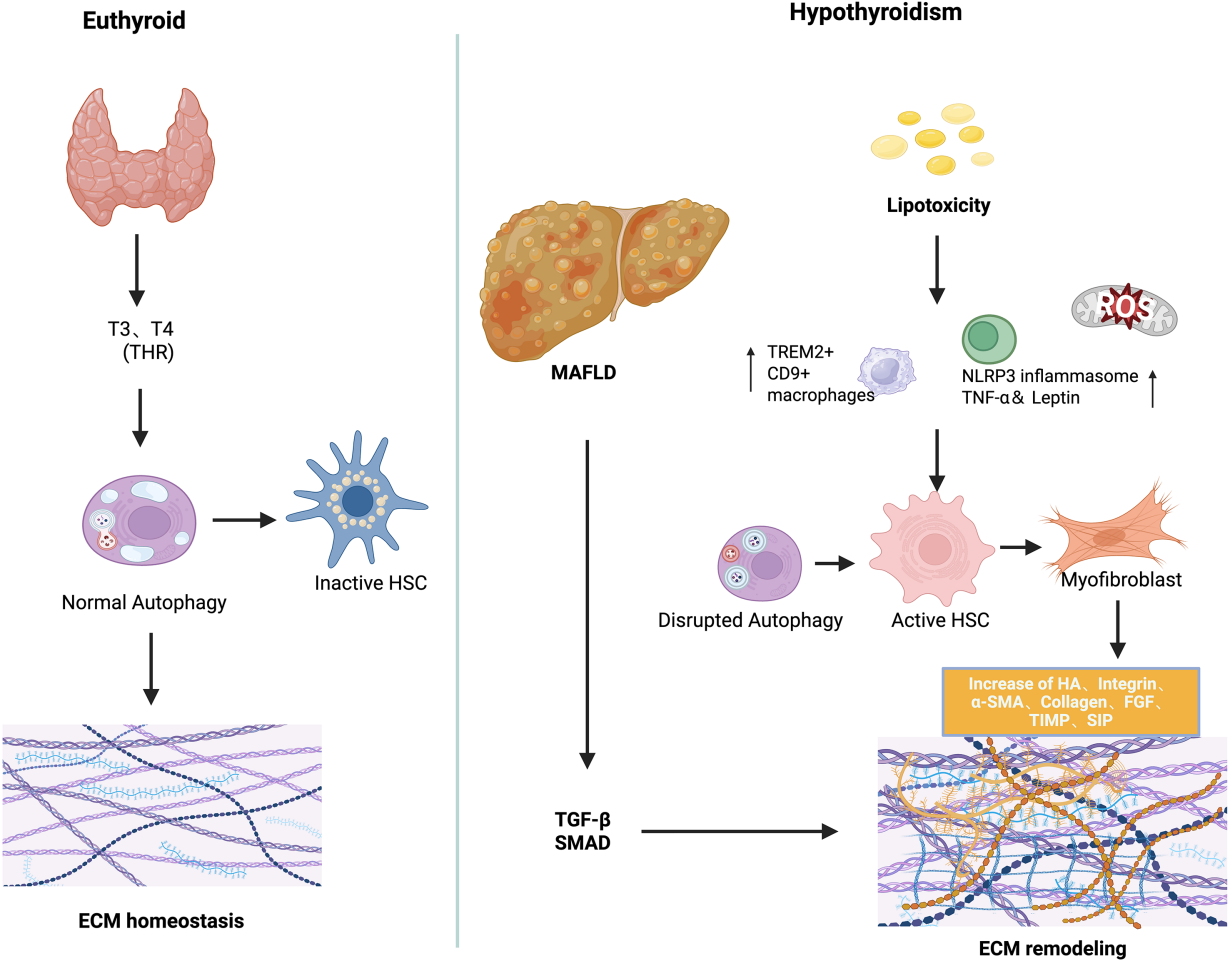
The mechanism of ECM remodeling in the pathogenesis of hypothyroidism and MAFLD.

Emerging evidence implicates hypothyroidism in the pathogenesis of MAFLD through dysregulated ECM remodeling (27). THs, particularly T3, play a crucial role in modulating HSC activation and fibrogenic signaling (81, 86–88). While general MAFLD studies provide insights into ECM dynamics, recent hypothyroidism-specific models demonstrate direct effects of thyroid hormone deficiency on ECM composition and stiffness. Under physiological conditions, hepatic ECM functions not only as a structural scaffold but also as a dynamic regulator of intercellular signaling, influencing lipid metabolism, immune homeostasis, and fibrogenesis (89). In hypothyroid states, excessive ECM synthesis—exacerbated by chronic inflammation—drives the progression from steatosis to fibrosis, cirrhosis, and ultimately hepatic failure. Sphingolipid accumulation within the ECM microenvironment, particularly ceramide-derived sphingosine-1-phosphate (S1P), plays a pivotal role in immune activation and fibrotic transformation (90). Elevated palmitate levels promote ceramide and S1P production, which binds to S1PR1–4 on macrophages, triggering HSC activation and differentiation into myofibroblast-like cells (91, 92). Experimental autoimmune thyroiditis (EAT) models have demonstrated that pharmacological blockade of S1P signaling ameliorates thyroid inflammation and reduces TSH levels, underscoring a bidirectional crosstalk between thyroid dysfunction and liver inflammation via ECM pathways (93). In hypothyroid states, reduced TH signaling impairs ECM turnover by increasing fibrillar collagen deposition, enhancing matrix stiffness, and attenuating matrix degradation, all of which accelerate hepatic fibrogenesis (27, 94, 95). Experimental hypothyroid models, including T3/T4 withdrawal and TH receptor (TRα/β) knockout mice, show spontaneous hepatic collagen accumulation and elevated expression of pro-fibrotic genes, confirming that TH deficiency alone can drive ECM remodeling. Through TRα expressed in HSCs, TH facilitates the reversion of activated myofibroblasts to a quiescent phenotype, promotes apoptotic clearance, and inhibits fibrogenic gene expression (27, 96). Consistent with these findings, experimental models demonstrate that T3 supplementation alleviates liver fibrosis by restoring mitochondrial biogenesis, enhancing β-oxidation of fatty acids, and normalizing autophagic flux. T3 supplementation in hypothyroid animal models attenuates TGF-β-induced fibrosis by reducing SMAD2 phosphorylation and transcriptional activity, restores mitochondrial biogenesis, enhances β-oxidation, and normalizes autophagic flux (11). Similarly, Alonso-Merino observed spontaneous hepatic collagen accumulation in TR-deficient mice, while T3 treatment ameliorated carbon tetrachloride (CCl_4_)-induced fibrosis, reinforcing the anti-fibrotic role of THs. In addition, T3 suppresses the expansion of TREM2^+^, CD9^+^ pro-fibrotic macrophages and inhibits the NLRP3 inflammasome, reducing hepatic inflammation (97). TH also upregulate matrix metalloproteinase (MMP) activity, promoting ECM degradation and tissue remodeling (98). In contrast, thyroxine (T4) may elicit pro-fibrotic effects through non-genomic signaling via integrin αvβ3, activating Rho-dependent pathways that induce α-SMA and p75 neurotrophin receptor (p75NTR) expression in HSCs (99). These context-dependent actions underscore the complexity of TH-mediated ECM regulation in liver fibrosis (28). Oxidative stress induced hepatocellular injury represents another potential mechanism linking thyroid dysfunction to liver fibrosis. Hypothyroidism may exacerbate this injury by disrupting systemic adipokine profiles—including tumor necrosis factor-alpha (TNF-α), Adiponectin, and leptin—thereby amplifying hepatic inflammation and fibrogenesis (100, 101). Beyond hormonal deficiency alone, the immunopathological characteristics of certain thyroid disorders may further exacerbate liver injury. In particular, autoimmune thyroid diseases—such as Hashimoto’s thyroiditis—have been increasingly associated with hepatic fibrogenesis. This association is hypothesized to arise from persistent systemic inflammation and dysregulated adipokine signaling intrinsic to autoimmune processes, which may synergistically promote hepatic inflammation and ECM accumulation in the context of thyroid hormone dysregulation (102, 103). RHAMM, a HA-binding receptor of the ECM, has emerged as a critical mediator of thyroid-liver crosstalk through its regulation of ECM remodeling (52). Our preclinical studies demonstrate that genetic ablation of RHAMM attenuates obesity-induced TSH elevation, mitigates hepatic oxidative stress, and modulates key metabolic signaling pathways in male mice. These findings indicate that RHAMM not only orchestrates ECM–cell interactions but also contributes to thyroid dysfunction and hepatic inflammation in hypothyroid states, potentially via modulation of antioxidant defense mechanisms such as Nqo1 (68). Although further validation in clinical settings is required, targeting RHAMM as an ECM receptor may represent a promising therapeutic strategy for obesity-related metabolic disorders, particularly those characterized by hypothyroidism-associated MAFLD (104). Collectively, these findings support a multifaceted role for TH in maintaining ECM homeostasis, and their dysregulation in hypothyroidism provides a mechanistic link to MAFLD development. At present, most evidence regarding ECM remodeling derives from studies in general MAFLD or hepatic fibrosis. Direct investigations under hypothyroid conditions are limited, with only a few studies using EAT models or TR-deficient mice suggesting a potential link between hypothyroidism and ECM alterations. The mechanism of ECM remodeling was shown in **Figure 2**.

### 3.1 Clinical evidence and disease heterogeneity

Thyroid dysfunction, particularly hypothyroidism, has emerged as a significant contributor to the development and progression of MAFLD (105). Obesity is frequently associated with abnormal fluctuations in TSH and TH levels—an endocrine imbalance strongly linked to various metabolic disorders, including type 2 diabetes and insulin resistance (58, 106). Clinical studies have demonstrated a positive correlation between TSH levels and visceral fat area, and hypothyroidism (both subclinical and overt) is more prevalent among obese individuals (107). In overt hypothyroidism, elevated TSH and reduced free T4 or T3 concentrations are frequently accompanied by the accumulation of lipotoxic intermediates and activation of pro-fibrotic pathways. Mechanistically, these effects are mediated in part through TSH receptor signaling in hepatocytes and HSCs, which promotes inflammatory and fibrogenic responses (108).

ECM remodeling is a pathological hallmark of hepatic fibrogenesis in MAFLD and plays a central role in linking thyroid dysfunction to progressive liver injury (109). In hypothyroid states, chronic metabolic stress enhances activation of HSC—the principal ECM-producing cells—leading to increased deposition of type I collagen and other matrix proteins (27). This process is further amplified by reduced activity of MMP and elevated expression of tissue inhibitors of TIMP, which collectively impair ECM degradation and perpetuate fibrotic remodeling (17, 110). HA, a major ECM component, is elevated in both hypothyroidism and advanced liver fibrosis and has emerged as a potential noninvasive biomarker of disease severity (111). Moreover, recent studies have shown that TSH receptor activation in preadipocyte-derived fibroblasts can directly stimulate HA synthesis (112). Given HA’s dual role as a matrix component and fibrosis biomarker, targeting its production may offer therapeutic value (113).

The heterogeneity of hypothyroidism significantly influences these ECM alterations and the clinical course of MAFLD. Clinical and preclinical data suggest that overt hypothyroidism exerts more pronounced effects on hepatic ECM composition and fibrotic signaling compared with subclinical forms, while autoimmune etiologies may further amplify inflammatory and fibrogenic responses (2, 114, 115). Coexisting metabolic disorders, including obesity and type 2 diabetes, further modulate ECM remodeling by promoting insulin resistance, lipid dysregulation, and low-grade inflammation (14, 116). In addition, sex-specific factors, particularly postmenopausal status in women, appear to affect ECM dynamics and MAFLD susceptibility, likely through estrogen-mediated regulation of hepatic metabolism and fibrogenic pathways (117, 118). Collectively, these findings underscore the importance of considering disease heterogeneity—including hypothyroidism subtype, autoimmune status, metabolic comorbidities, and sex differences-when evaluating ECM remodeling and MAFLD progression. Accounting for these variables in both clinical and preclinical studies may improve risk stratification, biomarker discovery, and the development of ECM-targeted therapeutic strategies.

From a therapeutic standpoint, several strategies aimed at restoring hepatic thyroid hormone action and correcting ECM dysregulation are under investigation. These include enhancing intracellular TH availability via modulation of transporters such as monocarboxylate transporter 8 (MCT8) and deiodinases (e.g., DIO1), as well as directly targeting ECM remodeling through upregulation of MMP activity, inhibition of HA synthesis, and blockade of integrin-mediated signaling pathways (119–121). Together, these findings underscore the clinical relevance of thyroid–ECM interactions in MAFLD and support the development of precision therapies for patients with comorbid thyroid dysfunction and liver fibrosis.

### 3.2 Pathological consequences

#### 3.2.1 Systemic fibrogenic amplification driven by ECM remodeling

Fibrotic diseases are a leading cause of global morbidity and mortality, histopathologically characterized by persistent ECM accumulation, especially of type I collagen (122). While traditionally considered organ-specific, fibrosis is increasingly recognized as a systemic pathology involving the liver, heart, lungs, and kidneys. Hypothyroidism has emerged as a key systemic amplifier of fibrotic remodeling, particularly in the context of MAFLD (2, 123). Shared fibrogenic pathways—including the TGF-β–HA/CD44–STAT3 axis—are activated across multiple organs in hypothyroid states (27). For instance, CD44 blockade reduces STAT3 activation and collagen production in atrial fibroblasts, HSCs, and pulmonary fibroblasts, underscoring a conserved ECM-mediated profibrotic mechanism (124). Hypothyroidism-induced metabolic and oxidative stress disrupts the homeostatic regulation of ECM turnover by MMP and their inhibitor (TIMP), promoting pathological cross-linking, stiffness, and matrix accumulatio (125). Lysyl oxidase (LOX)–mediated collagen cross-linking further increases ECM rigidity, which in turn activates integrin–FAK–YAP/TAZ and Wnt/β-catenin signaling pathways, and promotes epithelial–mesenchymal transition (EMT) and fibrogenesis (126). Additionally, fibrotic ECM suppresses antifibrotic miRNAs (e.g.,miR-29), perpetuating collagen synthesis (127). In the liver, impaired thyroid hormone signaling sustains HSC activation and α-SMA^+^ myofibroblast transformation, leading to excessive deposition of fibrillar collagens and matrix stiffening (128). This biomechanical stress alters hepatocyte polarity, suppresses regeneration, and induces apoptosis. These local hepatic alterations—when coupled with systemic inflammation, dysregulated adipokines (e.g., leptin, adiponectin), and circulating profibrotic cytokines (e.g., TGF-β, IL-13)—amplify fibrotic signaling across distant organs (123, 129). Thus, liver-derived ECM remodeling not only drives hepatic fibrosis but also acts as a nexus linking hypothyroidism to multi-organ fibrogenic progression.

#### 3.2.2 Disruption of ECM–cell communication

While systemic fibrosis reflects the macroscopic outcome of ECM dysregulation, the disruption of cell–matrix communication constitutes a pivotal microscopic mechanism that initiates and sustains tissue pathology (130). In hypothyroidism-associated MAFLD, aberrant ECM remodeling alters not only matrix composition but also its biomechanical properties, particularly stiffness. The accumulation of cross-linked collagens and fibronectin enhances tissue rigidity, which disrupts hepatocyte–matrix interactions. This mechanical stress impairs hepatocyte polarity, reduces regenerative capacity, and induces premature senescence or apoptosis (131). These biomechanical cues are sensed through integrins and focal adhesion complexes, activating downstream pathways such as FAK, RhoA/ROCK, and YAP/TAZ, which maintain HSC in a persistently activated state (132). Moreover, impaired ECM turnover due to MMP/TIMP imbalance further exacerbates matrix accumulation, forming a feed-forward loop (133). As a result, the ECM transitions from a supportive scaffold to a pathological regulator of hepatic injury and fibrogenesis.

#### 3.2.3 Immune rewiring and chronic inflammation

Chronic inflammation is a key pathological consequence of dysregulated ECM remodeling in hypothyroidism-associated MAFLD and serves as a major driver of hepatic fibrogenesis. This inflammatory response originates from hepatocellular injury and is perpetuated by the sustained activation of fibrotic signaling cascades (134). Macrophages are central to this process, orchestrating immune responses via cytokine secretion, phagocytosis of pathogens, apoptotic cells, and ECM debris, and facilitating tissue remodeling by releasing proteolytic enzymes and their inhibitors (135). In addition, macrophages interact with other immune and stromal cells—activating lymphocytes through antigen presentation and modulating fibroblast proliferation or apoptosis—thereby shaping the balance between repair and fibrosis (136). In hypothyroid conditions, ECM remodeling is significantly altered. Accumulation of glycosaminoglycans, particularly HA, aberrantly activates cell surface receptors such as CD44 and RHAMM (137). Persistent stimulation of these receptors amplifies pro-inflammatory signaling, promoting macrophage and T cell recruitment and sustaining hepatic inflammation (138). Fragmented ECM components like fibronectin and biglycan further act as damage-associated molecular patterns (DAMPs), engaging TLR2/4 on macrophages and enhancing cytokine release (139). Concurrently, TH deficiency impairs mitochondrial function and antioxidant defenses, disrupting immune-metabolic homeostasis (140). These alterations drive M1 polarization of hepatic macrophages via NLRP3 inflammasome activation, thereby reinforcing chronic inflammation (140, 141). Macrophage-derived MMPs and proinflammatory cytokines (e.g., IL-1β, IL-6, IL-12) further activate fibroblasts and pericytes, promoting ECM deposition (142). These interconnected events form a self-perpetuating feedback loop. Inflammatory mediators such as TGF-β stimulate HSCs via Smad and YAP/TAZ pathways, maintaining their activated, fibrogenic state. Meanwhile, increased ECM stiffness sustains mechanotransduction signaling in fibroblasts, reinforcing the cycle of immune activation, ECM remodeling, and fibrosis in hypothyroidism-associated MAFLD (143).

#### 3.2.4 Metabolic imbalance

In hypothyroidism-associated MAFLD, aberrant ECM remodeling plays a central role in disrupting hepatic metabolic homeostasis. The excessive accumulation of stiff, cross-linked ECM components impairs hepatocyte–matrix interactions and alters integrin-mediated mechanotransduction, thereby attenuating insulin signaling pathways (144). This mechanical dysfunction results in impaired PI3K-Akt activation and reduced translocation of glucose transporter GLUT2, ultimately leading to decreased hepatic glucose uptake and glycogen synthesis. Concurrently, fibrotic ECM deposition restricts the diffusion of oxygen and nutrients within hepatic lobules, generating localized hypoxia. This hypoxic environment inhibits mitochondrial β-oxidation via downregulation of carnitine palmitoyltransferase 1α (CPT1α), while promoting *de novo* lipogenesis through stabilization of hypoxia-inducible factor 1α (HIF-1α) and activation of SREBP-1c (145). Systemic hypothyroidism further exacerbates these metabolic disturbances by lowering circulating thyroid hormone levels and suppressing basal metabolic rate (146). Mechanistically, reduced expression of peroxisome PGC-1α and mitochondrial TFAM—key regulators of mitochondrial biogenesis and oxidative phosphorylation—compromises hepatic energy production and adaptability (147). Moreover, ECM-induced architectural remodeling may impair hepatic insulin clearance, a process that normally extracts approximately 50% of circulating insulin during the liver’s first pass (148, 149). Similar to conditions observed in cirrhosis and chronic hepatitis, reduced insulin clearance in fibrotic livers contributes to systemic hyperinsulinemia and insulin resistance (145). Collectively, these ECM-mediated alterations converge to promote hepatic metabolic inflexibility, creating a self-reinforcing cycle of mitochondrial dysfunction, IR, and lipid accumulation. This pathogenic loop accelerates the progression of hypothyroidism-associated MAFLD.

#### 3.2.5 ECM stiffening and hepatocarcinogenesis

In hypothyroidism-associated MAFLD, progressive ECM remodeling contributes not only to hepatic fibrogenesis but also to the initiation and progression of HCC (145). Excessive deposition of stiff, cross-linked ECM components including type I/III collagen, fibronectin, and HA alters the biomechanical properties of the hepatic microenvironment. This pathological stiffening disrupts hepatocyte polarity, promotes EMT, and facilitates malignant transformation. In hypothyroidism-associated MAFLD, persistent ECM remodeling not only underlies hepatic fibrogenesis but also functions as a mechanical and biochemical driver of hepatocarcinogenesis (150). Mechanistically, increased ECM stiffness activates integrin-mediated mechanotransduction pathways, notably the focal adhesion kinase (FAK), RhoA/ROCK, and YAP/TAZ signaling cascades (18, 151). These pathways converge on nuclear transcriptional programs that drive cell proliferation, inhibit apoptosis, and enhance stemness traits, ultimately predisposing hepatocytes to oncogenic reprogramming (152). Moreover, integrin-ECM interactions stimulate downstream PI3K/Akt and MAPK signaling, further enhancing hepatocyte survival, migration, and proliferative potential. Although certain integrin subtypes (e.g., β1-integrin) may exert context-dependent tumor-suppressive effects via p21/p27-mediated growth arrest, the prevailing outcome in fibrotic livers is a tumor-permissive microenvironment (153). Non-fibrillar ECM components such as laminin further contribute by modulating growth factor receptor activity and amplifying oncogenic signaling (150). Progressive liver fibrosis also enhances HSC and portal fibroblast activation through matrix rigidity, creating a positive feedback loop of fibrogenesis and oncogenesis (154). *In vitro* studies show that stiff collagen matrices impair hepatocyte differentiation and promote proliferation and chemoresistance via β1-integrin–mediated activation of FAK, ERK, Akt, and STAT3 pathways (155). Moreover, fibrotic vascular remodeling induces tissue hypoxia, stabilizing HIF-1α, which drives vascular endothelial growth factor (VEGF)-dependent angiogenesis and metabolic reprogramming toward aerobic glycolysis—hallmarks of tumor progression. Paradoxically, MMP, despite their degradative role, may also promote tumorigenesis by releasing sequestered growth factors and generating ROS via Rac1 signaling, thereby contributing to genomic instability (156). Clinically, patients with hypothyroidism and advanced hepatic fibrosis exhibit elevated liver stiffness values on elastography, which are strongly associated with increased HCC risk (97). These findings support a mechanistic link between thyroid dysfunction, fibrotic ECM remodeling, and hepatic carcinogenesis, positioning ECM stiffness as a critical pathogenic nexus in the transition from MAFLD to HCC (157).

## 4 Therapeutic strategies targeting ECM remodeling in MAFLD

Alterations in the composition of the ECM represent a key driver in the pathogenesis of MAFLD. Excessive production of ECM components, coupled with chronic inflammation, promotes the progression from steatosis to hepatic fibrosis, cirrhosis, and ultimately liver failure (90). Consequently, targeting the molecular mediators that govern ECM deposition and fibrogenesis has emerged as a promising therapeutic strategy. Recent advances have identified a diverse array of pharmacological agents capable of modulating ECM dynamics either directly, through inhibition of fibrogenic pathways and matrix-producing cells, or indirectly, by addressing the metabolic and inflammatory disturbances that drive ECM dysregulation and fibrotic progression.

Although MAFLD provides a broader and more inclusive diagnostic framework, current pharmacological strategies are largely based on evidence from NASH and liver fibrosis trials, which remain applicable in the context of progressive MAFLD. But their translation into routine practice necessitates rigorous evaluation of safety, durability of efficacy, and patient heterogeneity-factors that remain insufficiently addressed to date (20).

### 4.1 Liver-selective THRβ agonists for metabolic and fibrotic correction

Emerging pharmacological strategies have increasingly targeted THR signaling, particularly in the treatment of NASH and progressive MAFLD (158, 159). Experimental models have demonstrated that THR activation mitigates chemically induced liver fibrosis by reducing collagen deposition and α-SMA expression (96). Among THR isoforms, the β-subtype (THRβ)—predominantly expressed in hepatocytes—plays a central role in regulating mitochondrial bioenergetics, lipid metabolism, cholesterol homeostasis, and fatty acid oxidation (160). Liver-selective THRβ agonists have thus emerged as promising agents capable of reversing hepatic steatosis and attenuating fibrosis. The design of isoform- and hepatocyte-specific THRβ modulators offers a mechanistic basis for liver-targeted antifibrotic therapies, aiming to optimize efficacy while minimizing systemic adverse effects (8).

In clinical studies, Resmetirom (MGL-3196), the first THRβ agonist approved by the U.S. Food and Drug Administration (FDA) for patients with NASH and moderate to advanced fibrosis (F2–F3), has demonstrated favorable efficacy and safety profiles in late-stage clinical trials (161, 162). Similarly, VK2809 has shown significant reductions in hepatic fat content and was well tolerated in phase IIb trials (161). Several additional THRβ agonists are undergoing clinical evaluation, including ASC41 (phase II, China) (163, 164), HSK31679 (early study) (162), TERN-501 (165) (phase IIa), Sobetirome (GC-1, half before phase II) (164, 166), Eprotirome, Sobetirome, exhibited lipid-lowering effects in phase I trials under both single and multiple dosing regimens; however, its development was discontinued due to elevated liver enzyme levels, and no phase II trials were initiated (167). Eprotirome advanced to phase III for dyslipidemia but was withdrawn after preclinical studies revealed unexpected toxicities in animal models (168). These agents selectively activate THRβ in hepatocytes, hereby enhancing lipid metabolism, reducing hepatic steatosis, and suppressing inflammatory responses. Their demonstrated efficacy and relatively well-characterized safety profiles emphasize their potential as targeted therapies for MAFLD and NASH. However, long-term safety data, cost-effectiveness analyses, and the refinement of patient stratification strategies remain essential considerations prior to broader clinical implementation (8). In parallel, vitamin D receptor (VDR) agonists represent an additional nuclear receptor-based strategy with antifibrotic potential. These agents attenuate SMAD3 transcriptional activity, thereby reducing fibrogenic gene expression (96, 169). The selective VDR agonist compound 15a has been shown to suppress collagen deposition and fibrosis-related gene expression in murine models of hepatic fibrosis, without inducing hypercalcemia—a common side effect observed with Calcipotriol or 1,25(OH)_2_D_3_ (170). Beyond nuclear receptor-based strategies, other metabolic modulators also contribute to antifibrotic outcomes by mitigating hepatic steatosis and lipotoxicity, upstream drivers of fibrosis. Small molecule inhibitors targeting key enzymes of lipid synthesis—including firsocostat (Acetyl-CoA Carboxylase inhibitor), TVB-2640 (Fatty Acid Synthase inhibitor), and aramchol (Stearoyl-CoA Desaturase-1inhibitor) have shown efficacy in reducing hepatic fat accumulation and indirectly attenuating HSC activation. Furthermore, FGF21 analogues such as efruxifermin and pegozafermin improve insulin sensitivity and lipid profiles while suppressing fibrogenic pathways (171).

### 4.2 Direct anti-fibrotic agents targeting ECM remodeling

Targeting integrin-mediated activation of TGF-β has emerged as a promising antifibrotic approach within the broader strategy of ECM remodeling (172). Preclinical studies have demonstrated that inhibition of αv-containing integrins effectively attenuates fibrogenesis in models of NASH-related liver fibrosis (173). Among these, PLN-74809—a dual αvβ6/αvβ1 integrin inhibitor—has advanced to clinical trials in patients with liver fibrosis, with ongoing evaluation also in pulmonary fibrosis (174). Ganoderma lucidum polysaccharide (GLP), a 25 kDa water-soluble extract from sporoderm-removed spores, has demonstrated antifibrotic activity in CCl_4_-induced mouse models and TGF-β1-stimulated HSC-T6 cells. GLP attenuates HSC activation, collagen deposition, and α-SMA expression by suppressing TGF-β/Smad and TLR4/NF-κB signaling, modulating ECM–receptor interactions via downregulation of integrins (e.g., ITGA6, ITGA8), and influencing apoptosis and cell cycle regulators. While these findings suggest GLP as a promising ECM-targeted natural compound, its antifibrotic potential remains to be validated in clinical settings (175). ProAgio, a protein-based agent that targets a novel site on integrin αvβ3, induces selective apoptosis in activated HSC and capillarized liver sinusoidal endothelial cell. This results in reduced collagen crosslinking, restoration of sinusoidal architecture, and decreased intrahepatic angiogenesis, thereby reversing hepatic fibrosis and improving portal pressure and liver function (176). The hyaluronidase PH20 degrades HA, disrupting HA–CD44–TGF-β/SMAD2/3 signaling and demonstrating antifibrotic potential beyond its current ophthalmic applications (177). Although HA is a widely used serum biomarker of fibrosis, its specificity is limited, as it can be confounded by inflammation and other pathological conditions (178). Likewise, inhibitors targeting integrins remain at the stage of early clinical trials, with significant barriers to routine clinical translation. A critical evaluation of these biomarkers and therapeutic strategies highlights both their promise and their current limitations. Although these findings highlight integrin inhibitors as a viable class of ECM-targeted therapeutics, the majority of candidates remain at the preclinical or early clinical stage, and unresolved issues-including off-target toxicity, uncertainties in dose optimization, and limitations in delivery strategies pose substantial barriers to successful clinical translation (174).

I-BET151, a selective bromodomain and extraterminal domain (BET) inhibitor, suppresses HSC activation and ECM deposition by modulating pro-fibrotic gene transcription. Despite encouraging preclinical efficacy, no clinical trials have yet been initiated in liver fibrosis, highlighting the gap between mechanistic promise and translational feasibility (179). Similarly, inhibitors of lysyl oxidase-like enzymes (LOXL2/LOXL3), such as PXS-5153A, reduce collagen crosslinking and liver stiffness in preclinical settings (180). S1P signaling has been implicated in lipid-induced macrophage activation and HSC transdifferentiation. The S1P receptor modulator FTY720 mitigates hepatic injury, triglyceride accumulation, and fibrogenesis in murine MAFLD models by suppressing MMP2 and MMP9 expression and activity (181–183). However, its immunomodulatory properties and potential cardiovascular toxicity have constrained clinical development, highlighting the challenges of translating promising preclinical findings into safe and effective patient therapies (179, 183).

Galectin-3 inhibitors, such as belapectin, constitute another emerging class of antifibrotic agents that disrupt myofibroblast activation and collagen matrix organization. Although early-phase clinical trials have yielded variable outcomes, ongoing investigations continue to refine patient stratification strategies and evaluate combination regimens to enhance therapeutic efficacy (184). Preclinical studies indicate that T3 exerts antifibrotic effects by inhibiting the TGF-β/Smad signaling pathway. In hypothyroid states, the lack of T3-mediated inhibition may result in a relative increase in TGF-β/Smad activity, thereby promoting ECM accumulation and fibrosis (96, 185, 186). Thyroid dysfunction can amplify HA-CD44 signaling and collagen crosslinking, as evidenced by studies showing that hyaluronic acid promotes COX-2 expression in orbital fibroblasts from patients with thyroid-associated ophthalmopathy via CD44-mediated MAPK and NF-κB signaling (187). In this context, PH20, I-BET151, and PXS-5153A may indirectly counteract thyroid-related fibrosis by degrading ECM, inhibiting profibrotic signaling, or reducing collagen crosslinking. S1P receptor modulators and galectin-3 inhibitors could mitigate inflammation and hepatic stellate cell activation (188, 189), processes known to be exacerbated by hypothyroidism, further highlighting potential therapeutic avenues for hypothyroidism-associated MAFLD. In this context, antifibrotic agents may represent a promising strategy for hypothyroidism-associated MAFLD, as they could counteract enhanced fibrogenic signaling while also exerting direct effects on ECM remodeling. While these agents remain largely preclinical, their mechanistic alignment with thyroid-driven fibrosis strengthens their potential utility in hypothyroidism-associated MAFLD. Collectively, these findings underscore both the therapeutic potential and the uncertainties associated with translating single-agent antifibrotic therapies into routine clinical practice.

**Table 1** Summary of emerging therapeutic agents targeting ECM remodeling and associated pathways in metabolic MAFLD. Therapeutics are classified according to their primary mechanism of action, including antifibrotic, anti-inflammatory, lipid-modulatory effects. Development stages are based on current clinical or preclinical evidence.

**Table 1:** Therapeutic strategies targeting ECM remodeling and related pathways in MAFLD.

Therapeutic category	Representative agents	Mechanism	Development stage/indication
THRβ Agonists and Metabolic Modulators	MGL-3196; VK2809; ASC41; HSK31679; TERN-501; GC-1; Eprotirome	THRβ activation, improved lipid metabolism, mitochondrial function, inhibition of fibrosis	MGL-3196:FDA-approved;VK2809:phase IIb; ASC41:phase II; TERN-501: phase IIa; HSK31679: early study; GC-1:halt before phase II; Eprotirome: withdrawn after preclinical
	Compound 15a	VDR activation	Preclinical
	Firsocostat; TVB-2640; aramchol	Reduce hepatic steatosis and lipotoxicity	Firsocostat:phase II; TVB-2640:phase IIb; Aramchol: phase II
	Efruxifermin; Pegozafermin	FGF21 analogs	Preclinical
Direct Anti-fibrotic Agents	PLN-74809; ProAgio	Inhibition of integrins (αvβ6/αvβ1/αvβ3)	PLN-74809: clinical trials; ProAgio: Preclinical
	GLP	Suppression of TGF-β/Smad, TLR4/NF-κB	Preclinical
	I-BET151	BET protein modulation	Preclinical
	PXS-5153A	LOXL2/LOXL3 inhibition	Preclinical
	FTY720	Suppression of MMP2/9	Early trials, under study
	Belapectin	S1P signaling modulation	Phase II

## 5 Conclusion and perspectives

The intricate crosstalk between hypothyroidism and MAFLD is increasingly recognized as a multifactorial process involving endocrine dysfunction, metabolic dysregulation, and sustained ECM remodeling. This review underscores the pivotal role of TH signaling in modulating hepatic ECM dynamics and illustrates how ECM remodeling, in turn, amplifies fibrogenesis, oxidative stress, and insulin resistance, thereby accelerating MAFLD progression under hypothyroid conditions. The clinical and mechanistic evidence presented herein supports the concept that ECM remodeling functions not only as a downstream consequence but also as an active driver of disease synergy. Despite notable advances, several critical knowledge gaps remain. In particular, the temporal sequence and causal relationships among TH deficiency, ECM dysregulation, and hepatic injury require further elucidation through longitudinal studies and integrated multi-omics approaches (190). Moreover, emerging evidence suggests that immune–ECM interactions, the adipose–liver–thyroid axis, and gut microbiota may represent key modulators of this pathological interplay and merit deeper investigation (191, 192). From a translational perspective, targeting ECM components and associated signaling pathways—such as TGF-β, CD44/RHAMM, and THR modulators—represents a promising therapeutic avenue. However, future strategies must account for ECM heterogeneity (193), sex-specific responses, and the bidirectional dynamics of thyroid–liver axis regulation (194). Ultimately, interdisciplinary approaches bridging endocrinology, hepatology, and matrix biology will be essential for advancing precision diagnostics and tailored antifibrotic therapies in hypothyroidism-associated MAFLD.
